# Sex differences in dietary sodium evoked NCC regulation and blood pressure in male and female Sprague-Dawley, Dahl salt-resistant, and Dahl salt-sensitive rats

**DOI:** 10.1152/ajprenal.00150.2023

**Published:** 2024-05-30

**Authors:** Kiyoung Kim, Kayla M. Nist, Franco Puleo, James McKenna, Richard D. Wainford

**Affiliations:** ^1^Department of Pharmacology and Experimental Therapeutics, Whitaker Cardiovascular Institute, Boston University Chobanian and Avedisian School of Medicine, Boston, Massachusetts, United States; ^2^Department of Anatomy and Neurobiology, Boston University Chobanian and Avedisian School of Medicine, Boston, Massachusetts, United States; ^3^Division of Cardiology, Department of Medicine, Emory University School of Medicine, Atlanta, Georgia, United States

**Keywords:** blood pressure, NCC, NCC regulatory kinases, salt-sensitive hypertension, sex differences

## Abstract

Hypertension affects approximately one in two United States adults and sex plays an important role in the pathogenesis of hypertension. The Na^+^-Cl^−^ cotransporter (NCC), regulated by a kinase network including with-no-lysine kinase (WNK)1 and WNK4, STE20/SPS1-related proline alanine-rich kinase (SPAK), and oxidative stress response 1 (OxSR1), is critical to Na^+^ reabsorption and blood pressure regulation. Dietary salt differentially modulates NCC in salt-sensitive and salt-resistant rats, in part by modulation of WNK/SPAK/OxSR1 signaling. In this study, we tested the hypothesis that sex-dependent differences in NCC regulation contribute to the development of the salt sensitivity of blood pressure using male and female Sprague-Dawley (SD), Dahl salt-resistant (DSR), and Dahl salt-sensitive (DSS) rats. In normotensive salt-resistant SD and DSR rats, a high-salt diet evoked significant decreases in NCC activity, expression, and phosphorylation. In males, these changes were associated with no change in WNK1 expression, a decrease in WNK4 levels, and suppression of SPAK/OxSR1 expression and phosphorylation. In contrast, in females, there was decreased NCC activity associated with suppression of SPAK/OxSR1 expression and phosphorylation. In hypertensive DSS rats, the ability of females to suppress NCC (in opposition to males) via a SPAK/OxSR1 mechanism likely contributes to their lower magnitude of salt-sensitive hypertension. Collectively, our findings support the existence of sex differences in male versus female rats with NCC regulation during dietary salt intake involving suppression of WNK4 expression in male rats only and the involvement of SPAK/OxSR1 signaling in both males and females.

**NEW & NOTEWORTHY** NCC regulation is sex dependent. In normotensive male and female Sprague-Dawley and Dahl salt-resistant rats, which exhibit dietary Na^+^-evoked NCC suppression, male rats exhibit decreased WNK4 expression and decreased SPAK and OxSR1 levels, whereas female rats only suppress SPAK and OxSR1. In hypertensive Dahl salt-sensitive rats, the ability of females to suppress NCC (in opposition to males) via a SPAK/OxSR1 mechanism likely contributes to their lower magnitude of salt-sensitive hypertension.

## Graphical abstract

**Figure:**
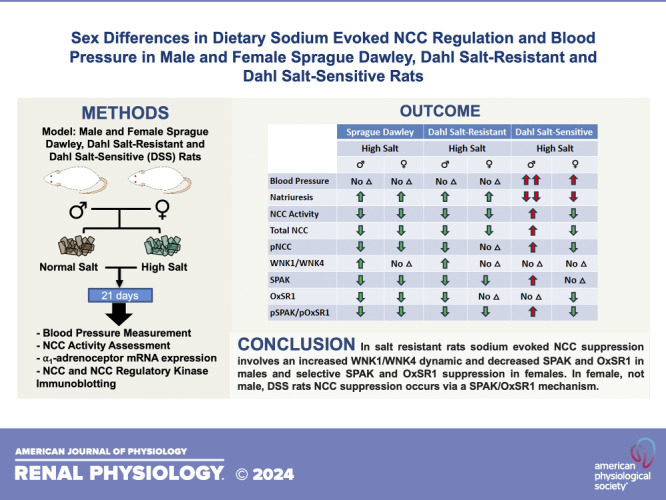

## INTRODUCTION

Hypertension, or high blood pressure, is a major risk factor for stroke, myocardial infarction, and chronic kidney disease (1, 2). Despite the availability of multiple classes of pharmacological interventions to lower blood pressure, hypertension is one of the leading causes of deaths worldwide (resulting in approximately 10 million deaths/yr) (1–3) and affects nearly one of two United States adults (4). Furthermore, sex plays an important role in the pathophysiology of hypertension. Men are more likely to develop hypertension at an earlier age and with greater severity than women. However, postmenopause, women’s risk for hypertension increases, and they are more likely to develop hypertension than men of the same age. Dietary Na^+^ intake is a modifiable risk factor for hypertension, as increased Na^+^ retention is a well-established precursor of hypertension risk (5). Dietary Na^+^ intake leads to an increased risk of hypertension in individuals who demonstrate the salt sensitivity of blood pressure, observed in approximately half of hypertensive individuals and a quarter of normotensive individuals (6, 7), and can result in the development of salt-sensitive hypertension (8). Given that United States adults consume approximately 3,400 mg of Na^+^ per day, which is in excess of the daily recommended 1,500 mg of Na^+^ per day by the American Heart Association (9, 10), salt-sensitive hypertension presents a major public health risk.

The kidneys are central to long-term blood pressure control through the regulation of Na^+^ reabsorption (11–13) and there is strong evidence that increased sympathetic nervous system activity can drive renal Na^+^ reabsorption and promote elevations in blood pressure. A key player in maintaining Na^+^ homeostasis is the Na^+^-Cl^−^ cotransporter (NCC), which is primarily located on the apical membrane of the distal convoluted tubule (DCT) of the kidneys (14, 15), whose expression and activity can be modulated by multiple factors including dietary salt and K^+^, elevations in sympathetic nervous system-mediated release of norepinephrine (NE), actions of the renin-angiotensin-aldosterone system, and local inflammation (16–18). NCC is regulated by a complex network of kinases that include with-no-lysine kinase (WNK)1 and WNK4, STE20/SPS1-related proline alanine-rich kinase (SPAK), and oxidative stress response 1 (OxSR1) (19–21). Dietary salt intake is known to modulate the activation and phosphorylation of NCC in both salt-sensitive and salt-resistant animal phenotypes (22–24), in part by modulation of the activation and phosphorylation of the WNK/SPAK/OxSR1 signaling network (15, 24–26).

The first major study to investigate the impact of sex on NCC expression showed that in female Sprague-Dawley (SD) rats loss of the female sex steroids following ovariectomy reduced NCC levels in the DCT, an effect prevented by 17β-estradiol administration (27), demonstrating an effect of the female sex steroids on NCC expression. Extending this study, Veiras et al. reported baseline sexual dimorphism in the expression of renal Na^+^ transporters in male and female SD rats (28). This study reported that, compared with males, female rats exhibit alterations in proteins relating to Na^+^ transport including increased Na^+^/H^+^ exchanger 3 (NHE3) phosphorylation, increased levels of total and phosphorylated NCC, and increased cleaved form of epithelial Na^+^ channel (ENaC) α- and γ-subunits. Female rats also exhibited a lower level of Na^+^-P_i_ cotransporter 2 (NaPi2) compared with male rats. Although several studies have explored the expression of NCC and renal Na^+^ transporters in the kidney, sex-dependent differences in the in vivo physiological activity of NCC and the regulatory kinases that impact NCC and how this impacts the salt sensitivity of blood pressure have not been extensively investigated.

We hypothesized that sex-dependent differences in the regulation of NCC contribute to the development of the salt sensitivity of blood pressure via a pathway that is reliant upon WNK/SPAK/OxSR1 signaling. To test this hypothesis, we used male and female SD rats and Dahl salt-resistant (DSR) and Dahl salt-sensitive (DSS) rats as a model to study the regulation of NCC and the development of salt sensitivity of blood pressure. In these animals, we evaluated blood pressure, NCC activity, NCC expression, and NCC regulation in response to 21-day normal or high dietary salt intake. Collectively, this study provides new insights into the sex-dependent regulation of NCC in the setting of salt resistance and the salt sensitivity of blood pressure. Our findings suggest the utility of developing sex-specific therapeutic interventions for hypertension and the salt sensitivity of blood pressure.

## MATERIALS AND METHODS

### Ethical Approval

All animal protocols were approved by the Institutional Animal Care and Use Committee (IACUC) under Protocol No. PROTO201800201 in accordance with the guidelines of Boston University and IPROTO202300000021 in accordance with the guidelines of Emory University and the National Institutes of Health *Guide for the Care and Use of Laboratory Animals* (8th ed.). All possible steps were taken to minimize pain and suffering, and euthanasia was conducted in accordance with approved IACUC protocols. Raw data will be made available upon reasonable request.

### Animals

Groups of male and female SD, DSR, and DSS rats (9–12 wk of age; Envigo, Indianapolis, IN) were used in these experiments. All animals were housed in the Boston University School of Medicine Laboratory Animal Science Center, a temperature (range: 20°C–26°C)- and humidity (range: 30–70%)-controlled facility, on a 12:12-h light-dark cycle. All rats were allowed tap water ad libitum and were randomly assigned to either a 21-day normal salt (NS) irradiated rodent diet [0.6% NaCl; Envigo Teklad, Teklad Global Diet No. 2918, 18% protein, 5% crude fat, 5% fiber, total K^+^ content 0.6%, total NaCl content 0.6% (174 mEq Na^+^/kg)] or an experimental high-salt (HS) irradiated diet [Envigo Teklad Diets, TD.03095, 19% protein, 5% crude fat, 3% dietary fiber, total K^+^ content 0.8%, total NaCl content 4% (678 mEq Na^+^/kg)] ad libitum.

### Surgical Procedures

On *day 21* of NS or HS dietary intake, rats were anesthetized with sodium brevital (20 mg/kg ip supplemented with 10 mg/kg iv as required). The left femoral artery and left femoral vein were cannulated with PE-50 tubing (Braintree Scientific, Braintree, MA) to measure mean arterial pressure (MAP) and to deliver intravenous infusions of saline or drugs, respectively. The bladder was cannulated with PE-240 tubing to facilitate urine collection (17, 22, 23). Rats received an intravenous infusion of isotonic saline (20 μL/min) during a 2-h recovery period to allow the animal to regain full consciousness and stable cardiovascular and renal excretory function (22, 23, 29–34). MAP was recorded continuously via the femoral artery cannula using BIOPAC data acquisition software (MP150 and AcqKnowledge 3.8.2; BIOPAC Systems) in conjunction with an external pressure transducer (P23XL; Viggo Spectramed) (22, 23, 29–34).

### Acute Experimental Protocols

The following protocols were performed consecutively in a single experimental period in each animal on *day 21* of NS or HS intake following femoral vein, artery, and bladder cannulation.

#### Cardiovascular function.

After the 2-h recovery period, baseline MAP was recorded continuously in conscious rats via the femoral artery cannula during the 1-h isotonic saline infusion period of the estimated in vivo NCC activity protocol. MAP values represent the average MAP during the entire 1-h isotonic saline infusion period (17, 22–24).

#### Estimated in vivo NCC activity.

Rats received an intravenous infusion of isotonic saline (20 μL/min) for a 1-h control period, a 1-h epithelial Na^+^ channel (ENaC) blockade period [amiloride (ENaC antagonist), 2 mg/kg bolus followed by 2 mg/kg/h at 20 μL/min], and a 1-h NCC blockade period during which ENaC blockade was maintained [hydrochlorothiazide (HCTZ; NCC antagonist), 2 mg/kg bolus followed by 2 mg/kg/h HCTZ + 2 mg/kg/h amiloride at 20 μL/min] (17, 22, 24, 35, 36). The use of amiloride allows for the isolation of NCC’s contribution to urinary Na^+^ excretion. MAP readings were obtained during the 1-h control period. Throughout the protocol, urine was collected via the bladder cannula in 10-min increments to assess urinary Na^+^ concentration. Estimated NCC activity was assessed as the peak natriuretic response (ΔUNaV; μEq/min) to HCTZ, calculated by subtracting average UNaV from the last two 10-min periods of ENaC blockade from maximum UNaV during NCC blockade (occurred within the first two 10-min periods of NCC blockade in all animals). Estimated ENaC activity was assessed as the peak ΔUNaV (μEq/min) to amiloride, calculated by subtracting average UNaV from the last two 10-min periods of control from maximum UNaV during ENaC blockade (occurred within the first two 10-min periods of ENaC blockade in all animals) (17, 22, 24, 35, 36). As a control, a separate group of male SD rats on NS intake received an intravenous infusion of HCTZ (NCC antagonist, 2 mg/kg bolus followed by 2 mg/kg/h HCTZ at 20 μL/min). In this study subgroup, we saw a significantly different, and attenuated, peak ΔUNaV to HCTZ of 5.2 ± 1.2 μEq/min.

### Kidney mRNA Expression

Kidneys were harvested from rats on a 21-day NS or HS diet immediately after decapitation and were immediately stored in RNAlater RNA stabilization solution (Cat. No. AM7021; Invitrogen) at −20°C. Approximately 100 mg of kidney cortex tissue was placed in 1 mL of QIAzol lysis reagent solution (Cat. No. 79306; QIAGEN, Hilden, Germany) with a stainless steel bead (Cat. No. 69989; QIAGEN) and homogenized using the QIAGEN TissueLyser II system at 30 Hz for 2 min. Samples were then left at room temperature for 5 min before being treated with 100 μL of gDNA Eliminator solution (Cat. No. 1062831; QIAGEN). To extract nucleic acids, 200 μL chloroform was added to each sample before samples were centrifuged at 17,000 *g* for 15 min at 4°C. Approximately 600 μL of the top aqueous layer was taken for mRNA purification using the QIAcube (QIAGEN) with RNeasy Plus Universal kit reagents and protocol (Cat. No. 73404; QIAGEN). Purified mRNA from each sample was converted to cDNA with a High Capacity RNA-to-cDNA kit (Cat. No. 4387406; Applied Biosystems). Real-time PCR was performed using a Viia7 thermal cycler (Applied Biosystems), RT^2^ SYBR Green ROX qPCR Mastermix (Cat. No. 330529; QIAGEN), and a custom Rat RT^2^ Profiler PCR array with propriety primer sequences (Cat. No. CAPR11754; QIAGEN). mRNA expression for all target genes was normalized to the expression of the following four housekeeping genes: *β-actin* (ACTB), *hypoxanthine phosphoribosyltransferase-1* (HPRT1), *lactate dehydrogenase A* (LDHA), and *ribosomal protein lateral stalk subunit P1* (RPLP1). The ΔΔC_T_ method (22) (where C_T_ is threshold cycle) was used to calculate mRNA expression changes between groups. Readouts are expressed as fold changes of NS + saline target gene expression. Analysis was carried out for each individual sample, as mRNA was not pooled (22).

### Kidney Protein Extract Preparation

Kidneys harvested from rats after decapitation and following completion of the acute experiments were immediately stored at –80°C (22, 24). Approximately 200 mg of kidney cortex tissue was homogenized on ice using a Potter-Elvehjem tissue grinder (Cat. No. 885510-0021; Kimble) in homogenizing buffer containing Halt Protease inhibitors cocktail (Cat. No. 78429; Thermo Scientific) and PhosSTOP phosphatase inhibitor (Cat. No. 04906845001; Roche) (22, 24). The homogenate was then centrifuged at 6,000 rpm for 15 min at 4°C. The supernatant was collected, and a bicinchoninic acid (BCA) assay was used to determine protein content. Prepared protein extracts were stored at –80°C prior to use in immunoblot experiments.

### Immunoblot Analysis

Kidney cortex protein extracts were loaded at 20 μg of total protein per lane. Nitrocellulose membranes (Cat. No. 1703932; Bio-Rad) were blocked in 5% blocking grade blocker (Cat. No. 170-6404; Bio-Rad) for 30 min and probed overnight at 4°C with primary antibodies in 0.1% PBS-Tween. Membranes were washed with 0.1% PBS-Tween and incubated for 2 h with secondary antibodies in 0.1% PBS-Tween at room temperature. Membranes were visualized using chemiluminescence (SuperSignal West Pico Plus, Cat. No. 34580; Thermo Scientific). Semiquantitative analysis was performed using ImageJ (version 1.52; National Institutes of Health) and protein expression (optical density/mm^2^) was normalized to total protein using Coomassie staining (Bio-Safe, Cat. No. 1610786; Bio-Rad) (24, 37). A series of gels was run using control protein standards to address antibody specificity and cross-reactivity. Protein standards for SPAK, OxSR1, and WNK4 were loaded on the same gel at 0.5 and 1 µg concentrations. The SPAK control was a purified (>95% pure) SPAK human recombinant protein, generated in baculovirus-infected Sf9 cells, that contains full-length human protein (96% homology with *Rattus norvegicus*) that has an N-terminal His tag with a predicted molecular weight of approximately 63 kDa (Recombinant Human SPAK, ab107696; Abcam). The OxSR1 control was a purified (>85% pure) OxSR1 human recombinant protein, generated in *Escherichia coli*, containing amino acids 1–527 of human OxSR1 protein (74% homology with *R. norvegicus*) that has an N-terminal His tag and a predicted molecular weight of approximately 60.4 kDa (OXSR1 Recombinant, Cat. No. 218418; United States Biological Life Sciences). The WNK4 control was a purified (94% pure) WNK4 human recombinant protein, generated in baculovirus-infected Sf21 cells, containing amino acids 1–444 of human WNK4 (90.32% homology with *R. norvegicus*) that has a GST tag and a predicted molecular weight of approximately 77 kDa [WNK4 (Human) Recombinant Protein, P5665; Abnova]. The NCC antibodies (total and phospho) used in this study were developed by Dr. Fenton’s laboratory and have been extensively validated by multiple groups and have been previously used by our laboratory generating results consistent with the current study (17, 22, 24). The WNK1 antibody used in this study has been previously validated with positive and negative controls (38) and has been previously published by our laboratory in rat kidney tissue (22, 24). A phosphatase (P9614; Sigma), which removes phosphate groups from serine, threonine, tyrosine, and histidine residues, was added to kidney lysates at a 1:20 ratio and incubated at 30°C for 30 min (20 µg protein), and untreated and phosphatase-treated samples were run next to each other. As shown in Supplemental Fig. S1, this significantly downregulated the signal detected by pNCC53. The antibodies and dilutions and standards and concentrations used are shown in Table 1 (24, 37).

**Table 1.** Antibodies and loading controls used for immunoblotting

Primary Antibody	Dilution	Source
NCC (sodium chloride cotransporter)	1:1,000	Millipore, Billerica, MA; Cat. No. ab3553; Lot No. 3532280
Phosphorylated NCC Thr^53^	1:1,000	PhosphoSolutions, Aurora, CO; Cat. No. p1311-53; Lot No. ks519b
WNK1 (with-no-lysine kinase 1)	1:200	Santa Cruz Biotechnology, Dallas, TX; Cat. No. sc-28897; Lot No. k2415
WNK4 (with-no-lysine kinase 4)	1:1,000	Novus Biologicals, Centennial, CO; Cat. No. NB600-284SS; Lot C
OxSR1 (oxidative stress response 1)	1:2,000	Abcam, Cambridge, MA; Cat. No. ab224248; Lot No. GR3257217-5
SPAK (STE20/SPS1-related proline-alanine-rich protein kinase)	1:500	Abcam, Cambridge, MA; Cat. No. ab79045; Lot No. 1004009-1
Anti-phospho SPAK (Ser^373^)/phosphor-OSR1 (Ser^325^)	1:500	MilliporeSigma, Burlington, MA; Cat. No. 07-2273; Lot No. 3851546
Anti-phospho OxSR1 (Thr^185^)	1:500	Abcam, Cambridge, MA; Cat. No. ab192803; Lot No. GR3269088-5

Secondary Antibody	Dilution	Source
HRP (horseradish peroxidase) donkey anti-rabbit IgG	1:2,000	Abcam, Cambridge, MA; Cat. No. ab16284; Lot No. GR3410837-2

Loading Control	Concentration	Source
WNK4	0.5 and 1 µg	Abnova, Taiwan; Cat. No. P5665; Lot No. 09CBS-0380E
SPAK	0.5 and 1 µg	Abcam, Cambridge, MA; Cat. No, ab107696; Lot No. 1061818-1
OxSR1	0.5 and 1 µg	United States Biological Life Sciences, Salem, MA; Cat. No. 218418; Lot No. L23090712

### ELISA

Kidneys harvested from rats after decapitation and following completion of the acute experiments were immediately stored at –80°C. Renal NE content was determined via ELISA per the manufacturer’s instructions (Cat. No. IB89537; Immuno-Biological Labs America, Minneapolis, MN) (36).

### Analytical Techniques

Urine volume was determined gravimetrically, assuming 1 g = 1 mL. Urine Na^+^ and K^+^ concentrations were assessed using flame photometry (model 943; Instrumentation Laboratories). Fractional excretion of Na^+^ (FE_Na_) was determined using standard techniques as previously described by our laboratory (24, 36).

### Statistical Analysis

Data are shown as means ± SD. Comparisons were made between NS and HS dietary salt intake within groups using a two-tailed Student’s *t* test, two-way ANOVA was used to assess differences between groups, and a post hoc test with Dunn–Šidák correction was used to evaluate variation among groups. Statistical analysis was conducted using GraphPad Prism (v. 9; GraphPad). Statistical significance is defined as *P* < 0.05.

## RESULTS

### Impact of High Dietary Na^+^ Intake on Blood Pressure and NCC Expression and Activity in Male and Female Salt-Resistant and Salt-Sensitive Rat Phenotypes

**Figure 1.**
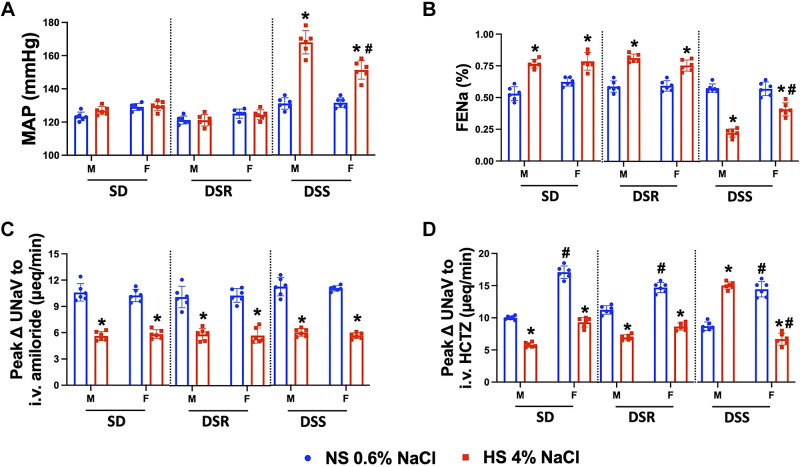
Impact of high dietary Na^+^ intake on blood pressure and NCC activity in male (M) and female (F) Sprague-Dawley (SD), Dahl salt-resistant (DSR), and Dahl salt-sensitive (DSS) rats. Mean arterial pressure (MAP; mmHg) (*A*), in vivo epithelial Na^+^ channel (ENaC) activity expressed as peak natriuretic response (ΔUNaV) to intravenous amiloride (2 mg/kg bolus, 2 mg/kg/h infusion) (*B*), in vivo Na^+^-Cl^–^ cotransporter (NCC) activity expressed as peak ΔUNaV to intravenous hydrochlorothiazide (HCTZ; 2 mg/kg bolus, 2 mg/kg/h infusion) (*C*), and fractional excretion of Na^+^ (FE_Na_; %) (*D*) in 3-mo-old male and female SD, DSR, and DSS rats fed a 21-day normal salt (NS; 0.6% NaCl) or high-salt (HS; 4% NaCl) diet are shown. Values are means ± SD; *n* = 6 animals per group. Differences in peak ΔUNaV, MAP, and FE_Na_ within each group between NS and HS diets were determined using a Student’s *t* test. Differences between groups (male vs. female) were determined using two-way ANOVA to assess differences between groups, and a post hoc test with Dunn–Šidák correction was used to evaluate variation among groups. *P < 0.05 vs. the respective NS group. #*P* < 0.05 vs. the respective male group.

When challenged with a HS diet, male and female SD and DSR rats increase FE_Na_ and maintain normotension (Fig. 1, *A* and *B*). In contrast, male and female DSS rats decreased FE_Na_ and exhibited salt sensitivity of blood pressure, changes that were of a significantly higher magnitude in male versus female DSS rats (Fig. 1, *A* and *B*). Males and females of all three rat strains exhibited dietary Na^+^-evoked suppression of estimated ENaC activity (expressed as peak ΔUNaV to amiloride) (Fig. 1*C*). Furthermore, male and female SD and DSR rats and female DSS rats exhibited dietary Na^+^-evoked suppression of in vivo estimated NCC activity (expressed as peak ΔUNaV to HCTZ) (Fig. 1*D*). Critically, male DSS rats maintained on a HS diet exhibited a significant increase in in vivo estimated NCC activity (Fig. 1*D*). In SD, DSR, and DSS rats, we observed no HS evoked alterations in baseline Na^+^ and K^+^ excretion during the in vivo estimated NCC activity assay (Table 2).

**Table 2.** Baseline renal excretory parameters during acute renal sodium transporter assay

Study Group	Baseline Na^+^ Excretion, μEq/min	Baseline K^+^ Excretion, μEq/min	Baseline Urine Output, μL/min
Male SD NS	2.25 ± 0.31	2.12 ± 0.38	22.7 ± 4.1
Female SD HS	2.39 ± 0.26	2.18 ± 0.31	20.4 ± 3.7
Male DSR NS	2.44 ± 0.34	2.12 ± 0.44	21.1 ± 3.4
Female DSR HS	2.31 ± 0.32	2.17 ± 0.32	21.6 ± 4.7
Male DSS NS	2.49 ± 0.25	2.11 ± 0.35	22.1 ± 3.3
Female DSS HS	2.51 ± 0.34	2.01 ± 0.28	20.9 ± 3.8

Data are expressed as means ± SD (*n* = 6 animals/group). Baseline sodium excretion (μEq/min), baseline potassium excretion (μEq/min), and baseline urine output (μL/min) during the control isotonic saline infusion period of the acute renal sodium transporter activity assay in conscious naive male and female SD, DSR, and DSS rats maintained on either a normal (NS; 0.6% NaCl) or a high-salt (HS; 4% NaCl) diet for 21 days. DSR, Dahl salt-resistant; DSS, Dahl salt-sensitive; SD, Sprague-Dawley.

### Impact of High Dietary Na^+^ Intake on Renal NE Levels and α_1_-Adrenoceptor Subtype Levels

**Figure 2.**
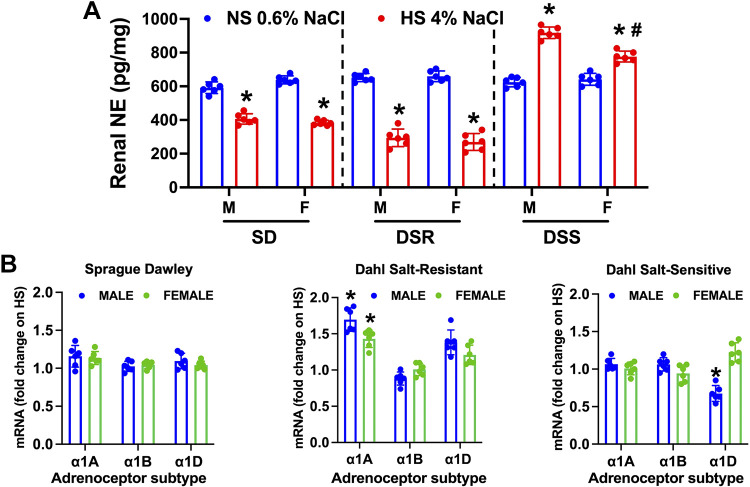
Impact of high dietary Na^+^ intake on renal norepinephrine (NE) content and mRNA expression of α_1_-adrenoceptor subtypes in male (M) and female (F) Sprague-Dawley (SD), Dahl salt-resistant (DSR), and Dahl salt-sensitive (DSS) rats. Renal NE content (pg/mg) (*A*) and α_1_-adrenoceptor subtypes [mRNA fold change on high salt (HS)] (*B*) in 3-mo-old male and female SD, DSR, and DSS rats fed a 21-day normal salt (NS; 0.6% NaCl) or HS (4% NaCl) diet are shown. Values are means ± SD; *n* = 6 animals per group. Differences in renal NE and mRNA expression between groups (male vs. female) were determined using two-way ANOVA, and a post hoc test with Dunn–Šidák correction was used to evaluate variation among groups. *P < 0.05 vs. the respective NS group. #*P* < 0.05 vs. the respective male group.

When challenged with an HS diet, male and female SD and DSR rats decreased sympathetic outflow to the kidney, as assessed by renal NE content, to a similar magnitude showing no sex or strain differences (Fig. 2*A*). In contrast, male and female DSS rats increased renal NE content in response to HS intake and exhibited a sex-dependent response with males exhibiting a significantly higher increase in renal NE content than females (Fig. 2*A*). The analysis of mRNA expression of α_1_-adrenoceptor subtypes revealed strain and sex differences. In contrast to SD rats, in which HS intake did not impact α_1_-adrenoceptor subtype mRNA expression, both male and female DSR rats exhibited a significant increase in α_1A_ mRNA levels. Female DSS rats exhibited no changes in α_1_-adrenoceptor subtype expression, whereas male DSS rats exhibited a decrease in α_1D_ levels (Fig. 2*B*).

### Impact of High Dietary Na^+^ Intake on NCC Expression and Phosphorylation in Male and Female Salt-Resistant and Salt-Sensitive Phenotypes

**Figure 3.**
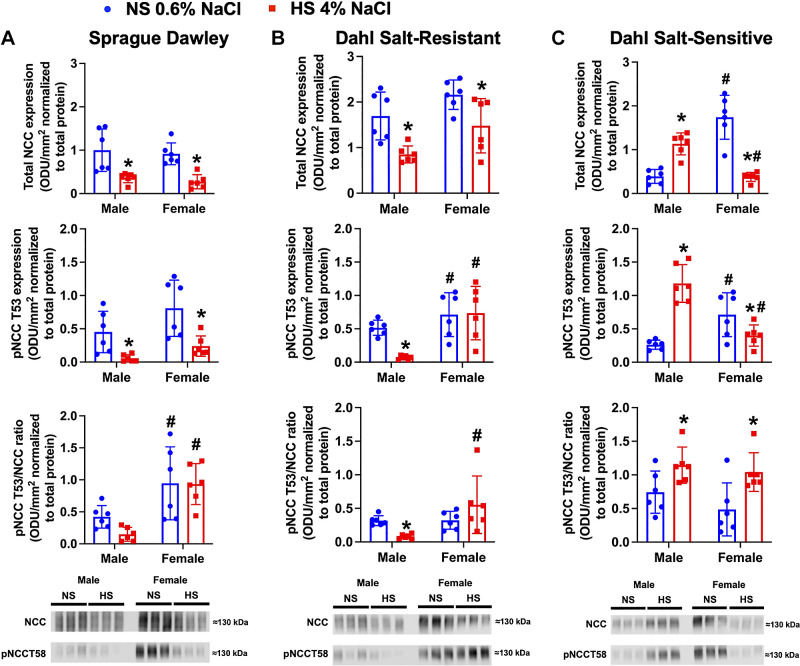
Impact of high dietary Na^+^ intake on total Na^+^-Cl^–^ cotransporter (NCC) expression and phosphorylation in male and female Sprague-Dawley (SD), Dahl salt-resistant (DSR), and Dahl salt-sensitive (DSS) rats. Total NCC protein expression and NCC phosphorylation (pNCCT53) expressed as fold change and representative immunoblots for total NCC and pNCCT53 in 3-mo-old male and female SD (*A*), DSR (*B*), and DSS rats (*C*) fed a 21-day normal salt (NS; 0.6% NaCl) or high-salt (HS; 4% NaCl) diet are shown. Values are means ± SD; *n* = 6 animals per group. Protein expression is shown as optical density units (ODU)/mm^2^ normalized to total protein. Differences in protein expression within each group between NS and HS diets were determined using a Student’s *t* test. Differences in protein expression between groups (male vs. female) were determined using two-way ANOVA, and a post hoc test with Dunn–Šidák correction was used to evaluate variation among groups. *P < 0.05 vs. the respective NS group. #*P* < 0.05 vs. the respective male group.

HS intake reduced in vivo NCC activity in normotensive male and female SD rats (Fig. 1*D*) and was paralleled by HS evoked suppression of both total NCC expression and NCC Thr^53^ phosphorylation normalized to Coomassie blue (Fig. 3*A* and Supplemental Fig. S1). However, there was a sex difference with female SD rats exhibiting no change in the overall pNCC/NCC ratio following HS.

In male and female DSR rats, we observed suppression of total NCC expression following a HS diet consistent with in vivo NCC activity (Figs. 1*D* and 3*B* and Supplemental Fig. S2). Although male DSR rats suppressed NCC Thr^53^ phosphorylation in response to a HS diet, which resulted in a reduced pNCC/NCC ratio, female DSR rats failed to suppress NCC Thr^53^ phosphorylation in response to an HS diet, resulting in no change in the total pNCC/NCC ratio (Fig. 3*B*).

Confirming our in vivo NCC activity data (Fig. 1*D*), we observed a dietary Na^+^-evoked increase in both total NCC expression and NCC Thr^53^ phosphorylation normalized to Coomassie blue in male DSS rats (Fig. 3*C* and Supplemental Fig. S3). In contrast, we observed significant suppression of total NCC expression and NCC Thr^53^ phosphorylation following a HS diet in female DSS rats (Fig. 3*B*). However, despite the differential sex-dependent effects of HS on total and phosphorylated NCC in male and female rats, both sexes exhibited an increased pNCC/NCC ratio in response to an HS diet.

### Impact of High Dietary Na^+^ Intake on WNKs in Male and Female Salt-Resistant and Salt-Sensitive Phenotypes

**Figure 4.**
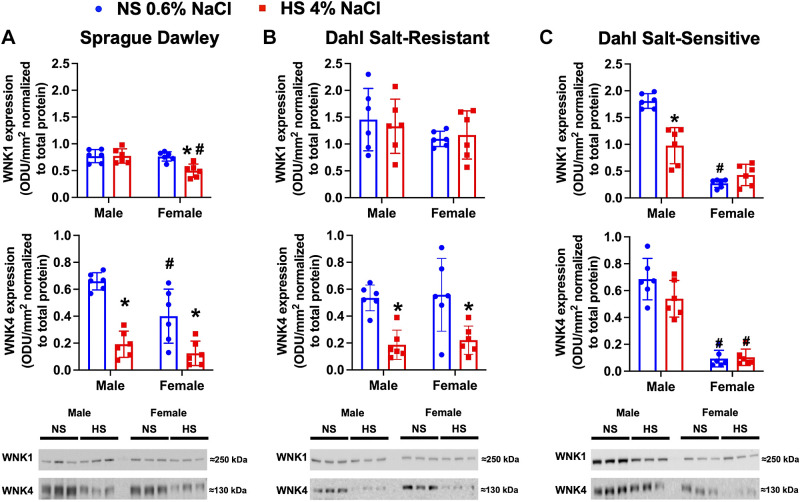
Impact of high dietary Na^+^ intake on with-no-lysine kinase (WNK)1 and WNK4 expression in male and female Sprague-Dawley (SD), Dahl salt-resistant (DSR), and Dahl salt-sensitive (DSS) rats. Ttotal WNK1 and WNK4 protein expression expressed as fold change and representative immunoblots in 3-mo-old male and female SD (*A*), DSR (*B*), and DSS (*C*) rats fed a 21-day normal salt (NS; 0.6% NaCl) or high-salt (HS; 4% NaCl) diet are shown. Values are means ± SD; *n* = 6 animals per group. Protein expression is shown as optical density units (ODU)/mm^2^ normalized to total protein. Differences in protein expression within each group between NS and HS diets were determined using a Student’s *t* test. Differences in protein expression between groups (male vs. female) were determined using two-way ANOVA, and a post hoc test with Dunn–Šidák correction was used to evaluate variation among groups. *P < 0.05 vs. the respective NS group. #*P* < 0.05 vs. the respective male group.

In male SD and DSR rats, we observed no impact of a HS diet on the protein expression of WNK1 but observed profound HS evoked suppression of WNK4 protein expression (Fig. 4). Demonstrating sex differences in female SD rats, we observed HS evoked suppression of WNK1 and WNK4, and in female DSR rats, we observed dietary Na^+^-evoked suppression of WNK4.

In contrast to SD and DSR rats, male DSS rats had HS intake-evoked suppression of WNK1 with no significant change in WNK4 expression. Demonstrating a sex difference, female DSS rats expressed significantly lower levels of WNK1 and WNK4 compared with male DSS rats on NS intake and did not exhibit HS-evoked changes in WNK1 or WNK4 expression.

### Impact of High Dietary Na^+^ Intake on SPAK/OxSR1 in Male and Female Salt-Resistant and Salt-Sensitive Phenotypes

**Figure 5.**
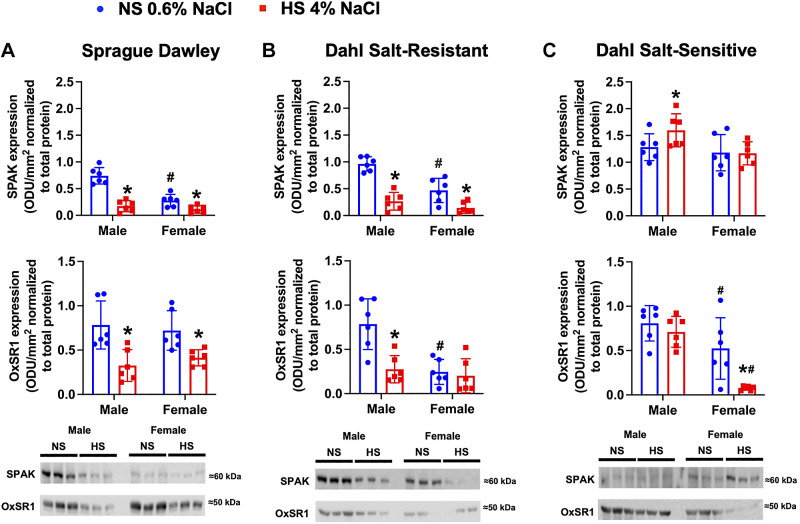
Impact of high dietary Na^+^ intake on STE20/SPS1-related proline alanine-rich kinase (SPAK) and oxidative stress response 1 (OxSR1) expression in male and female Sprague-Dawley (SD), Dahl salt-resistant (DSR), and Dahl salt-sensitive (DSS) rats. Total SPAK and OxSR1 protein expression expressed as fold change and representative immunoblots in 3-mo-old male and female SD (*A*), DSR (*B*), and DSS (*C*) rats fed a 21-day normal salt (NS; 0.6% NaCl) or high-salt (HS; 4% NaCl) diet are shown. Values are means ± SD; *n* = 6 animals per group. Protein expression is shown as optical density units (ODU)/mm^2^ normalized to total protein. Differences in protein expression within each group between NS and HS diets were determined using a Student’s *t* test. Differences in protein expression between groups (male vs. female) were determined using two-way ANOVA, and a post hoc test with Dunn–Šidák correction was used to evaluate variation among groups. *P < 0.05 vs. the respective NS group. #*P* < 0.05 vs. the respective male group.

Baseline levels of SPAK and OxSR1 were lower in female SD and DSR rats on an NS diet compared with their respective male rats within strain (Fig. 5, *A* and *B*). In response to an HS diet, male and female SD and male DSR rats downregulated SPAK and OxSR1 protein expression (Fig. 5, *A* and *B*). In contrast, female DSR rats suppressed only SPAK in response to HS intake (Fig. 5, *A* and *B*). DSS rats did not exhibit sex differences in basal SPAK and OxSR1 expression. However, highlighting sex differences, male DSS rats increased SPAK with no change in OxSR1 in response to HS intake, whereas female DSS rats had no change in SPAK but exhibited significant downregulation of OxSR1 only (Fig. 5*C*).

**Figure 6.**
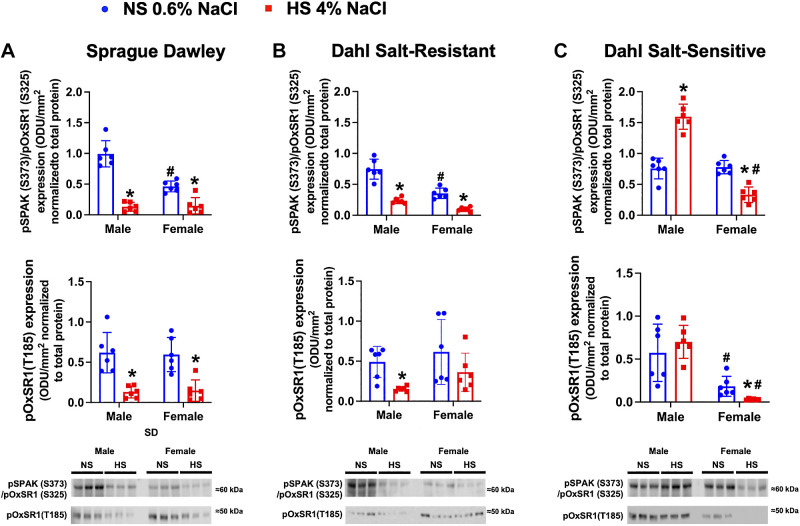
Impact of high dietary Na^+^ intake on expression of phosphorylated STE20/SPS1-related proline alanine-rich kinase (SPAK) and oxidative stress response 1 (OxSR1) expression in male and female Sprague-Dawley (SD), Dahl salt-resistant (DSR), and Dahl salt-sensitive (DSS) rats. Total pSPAK (S373)/pOxSR1 (S325) expression and pOxSR1 (T185) expression SPAK and OxSR1 protein expression expressed as fold change and representative immunoblots in 3-mo-old male and female SD (*A*), DSR (*B*), and DSS (*C*) rats fed a 21-day normal salt (NS; 0.6% NaCl) or high-salt (HS; 4% NaCl) diet are shown. Values are means ± SD; *n* = 6 animals per group. Protein expression is shown as optical density units (ODU)/mm^2^ normalized to total protein. Differences in protein expression within each group between NS and HS diets were determined using a Student’s *t* test. Differences in protein expression between groups (male vs. female) were determined using two-way ANOVA, and a post hoc test with Dunn–Šidák correction was used to evaluate variation among groups. *P < 0.05 vs. the respective NS group. #*P* < 0.05 vs. the respective male group.

Phosphorylated SPAK/OxSR1 expression at serine residues (pSPAK Ser^373^/pOxSR1 Ser^325^) on a NS diet was significantly lower in female SD and DSR rats compared with male SD and DSR rats (Fig. 6, *A* and *B*). In response to an HS diet, male and female SD and DSR rats downregulated phosphorylation of SPAK and OxSR1, which was assessed as a collective decrease in the phosphorylation of SPAK and OxSR1 at serine and threonine sites (Fig. 6, *A* and *B*).

In DSS rats, there was no difference in baseline serine SPAK/OxSR1 phosphorylation; however, female DSS rats exhibited lower pOxSR1 Thr^185^ phosphorylation on NS intake (Fig. 6*C*). Male DSS rats exhibited increased serine SPAK/pOxSR1 phosphorylation and a failure to suppress pOxSR1 Thr^185^ phosphorylation (Fig. 6*C*). In contrast, female DSS rats exhibited dietary Na^+^-evoked suppression of phosphorylation of SPAK and OxSR1, as observed in SD and DSR rats (Fig. 6*C*).

## DISCUSSION

The major findings of this study are that in response to high dietary salt intake, the regulation of NCC exhibits sex and strain dependency. Specifically, *1*) there are sex-dependent differences in the signaling mechanisms underlying the suppression of NCC activity in normotensive male and female salt-resistant SD and DSR rats with male rats exhibiting decreased WNK4 expression and decreased SPAK and OxSR1, whereas female rats only suppress SPAK and OxSR1; *2*) the development of salt-sensitive hypertension in male, but not female, DSS rats involves the failure to suppress NCC activity and dysregulation of the WNK/SPAK/OxSR1 signaling network; and *3*) the ability of female DSS rats to suppress NCC occurs via a SPAK/OxSR1 mechanism.

Our finding of elevated NCC activity and expression in female versus male rats (SD, DSR, and DSS strains) is supported by recent evidence of increased NCC levels in female versus male C57BL6/J mice (40) and SD rats (28). We speculate that the differential higher expression of NCC in female versus male rats is driven by the presence of female sex steroids (27, 41). Despite the elevated baseline activity and expression of NCC, both normotensive male and female SD and DSR rats exhibit dietary Na^+^-evoked suppression of NCC activity, total NCC protein levels, and increased FE_Na_ (partly reflective of the steep slope of the pressure-natriuresis relationship in these salt-resistant phenotypes) (30, 36). These findings are consistent with our previous studies in male SD and DSR rats (17, 22, 24) and the study of Veiras et al. in male and female SD rats (28). Furthermore, these findings are supported by evidence in male DSR rats that an HS diet reduces ΔUNaV to NCC antagonism (42) and extend these findings to female SD and DSR rats. Significantly, there was no impact of HS intake on NCC phosphorylation in female DSR rats, reflecting a sex difference in DSR rats and a strain difference between female SD and DSR rats. This suggests that in female DSR rats, suppression of total NCC levels, and therefore a reduction in the total amount of NCC that can be phosphorylated and active, is sufficient to facilitate the observed reduction in in vivo NCC activity. Collectively, these data suggest that dietary Na^+^-evoked NCC downregulation in salt-resistant rat phenotypes is a conserved response to maintain Na^+^ balance and normotension.

Replicating our prior data (24), male DSS rats fed an HS diet exhibited significant increases in NCC activity, expression, and phosphorylation and decreased FE_Na_, factors that contribute to the observed salt sensitivity of blood pressure and an attenuated pressure-natriuresis response. Our finding that both male and female DSS rats exhibit HS-evoked suppression of estimated ENaC activity is consistent with our prior finding in male DSS rats (24). However, given the indirect nature of this finding, and the preexisting literature supporting a role of ENaC in DSS rat hypertension, we do not believe that this implies a lack of involvement of ENaC in the pathophysiology of DSS rat hypertension. In agreement with prior findings (43, 44), we observed an attenuated magnitude of hypertension and salt sensitivity of blood pressure, of ∼15 mmHg, in female DSS rats. The observed HS-evoked suppression of NCC activity in female DSS rats likely contributes to this observed sex difference in blood pressure. In addition, we speculate that the expression of additional Na^+^ transporters present within the kidney may be altered in a sex-dependent fashion in female DSS rats to aid in maintaining Na^+^ homeostasis.

Our present observations of suppression of renal sympathetic tone in salt-resistant versus salt-sensitive rat phenotypes are consistent with previous studies linking sympathetic outflow to NCC stimulation in rodent models (17, 22, 24, 45, 46). Further supporting a role for renal α_1_-adrenoceptor signaling in mediating sympathetic signaling at the level of the kidney to impact NCC regulation, as previously reported (17, 22, 24, 45, 46) in response to HS intake, we observed an increase in α_1A_-adrenoceptor mRNA expression in male and female DSR rats and sex-specific downregulation of α_1D_-adrenoceptor mRNA expression in male DSS rats. Given that α_1D_-adrenoceptor knockout mice exhibit attenuated salt-sensitive hypertension (47), we speculate that the observed downregulation of α_1D_-adrenoceptor mRNA in male DSS rats reflects a compensatory mechanism to counter the magnitude of the salt sensitivity of blood pressure. However, the roles of the α_1A_- and α_1D_-adrenoceptors in the regulation of NCC are currently unknown, and future studies are required to delineate the role of α_1_-adrenoceptor subtypes.

In response to HS intake, male SD and DSR rats exhibit no change in WNK1 and a significant downregulation of WNK4. We acknowledge that in our prior publication (24) we observed a small, but significant, increase in WNK1 expression following HS intake in male DSR rats. However, the lack of increase observed in this study may reflect either a lot of variation in antibodies or the analysis of a membrane preparation versus a crude kidney preparation in this study. Replicating our prior findings (24), there was no decrease in WNK4 expression in male DSS rats. Highlighting a clear sex difference, we observed no dietary salt evoked alteration in the relative expression of WNK1 to WNK4 in female SD, DSR, or DSS rats. These data suggest the roles of WNK1 and WNK4 in response to dietary salt intake are sex dependent in both salt-resistant and salt-sensitive phenotypes. Furthermore, these data suggest a sex-dependent impact of WNK1 and WNK4 on NCC regulation and the salt sensitivity of blood pressure. Our data suggest that dynamic dietary Na^+^-evoked alterations in the relative expression levels of WNK1 and WNK4 (i.e., a decrease in WNK4) play a role in the phenotype of salt resistance in male, but not female, rats and impairments in this response contribute to the salt sensitivity of blood pressure. It remains to be established how the alteration in the expression levels of WNK1 and WNK4 impact NCC phosphorylation, and it remains to be established if WNK1 can modify WNK4. The WNK1 antibody used targets the C-terminus and can theoretically recognize both WNK1 isoforms: kidney-specific WNK1 (KS-WNK1), which lacks a functional kinase domain, and full-length long WNK1 (LWNK1). As such, we expressed our data as total WNK1 as we were unable to determine which isoform of WNK1 was altered in the current study. We believe that our protein expression data likely reflect alterations in KS-WNK1 given that L-WNK1 transcripts are present at low abundance in contrast to KS-WNK1 transcripts, which are highly expressed, almost exclusively, in the DCT, where they are ∼80 times more abundant than L-WNK1 (48, 49). Given that several previous studies have demonstrated that KS-WNK1 is able to lower NCC activity, by forming a complex with L-WNK1 to inhibit its ability to influence NCC in *Xenopus* oocytes (50), in KS-WNK1 transgenic mouse renal cortex and total and phosphorylated NCC levels are reduced (51) and KS-WNK1 knockout mice have increased NCC expression and hypertension (51, 52). These studies are consistent with KS-WNK1 being an inhibitor of NCC. Given that our data suggest that decreased WNK4 levels and maintained levels of WNK1 correlate with decreased NCC activity, we speculate that our expression data reflect increased KS-WNK1 expression and the actions of KS-WNK1 to suppress NCC activity and phosphorylation. In contrast, there is evidence that supports the hypothesis that KS-WNK1 can stimulate NCC (53). Moreover, we acknowledge that the observed changes in WNK1/4 abundance in response to HS intake may not reflect the expression of these kinases in the DCT. Therefore, future studies are needed to address DCT-specific expression of WNKs and their effects on NCC regulation.

In relation to the baseline expression of SPAK and OxSR1, there is a clear sex difference with female SD and DSR rats having lower levels of total SPAK and serine phosphorylated SPAK/OxSR1. However, there is no sex difference in the response to dietary salt intake in SD rats. As observed in SD rats, male DSR rats suppress SPAK and OxSR1 expression and phosphorylation during HS intake, but, demonstrating strain and sex differences, female DSR rats suppress only total SPAK and serine phosphorylated SPAK/OxSR1. Clear sex differences are observed in DSS rats with male DSS rats exhibiting an increase in total SPAK and SPAK/OxSR1 serine phosphorylation and absence of suppression of OxSR1, data that correlate with increased NCC activity in male DSS rats. In contrast, in female DSS rats, which suppress NCC activity during HS, there is suppression of OxSR1 and SPAK/OxSR1 phosphorylation. Therefore, a major finding of this study is the SPAK/OxSR1-mediated regulation of NCC in female rats independently from alterations in the expression of WNK1 and WNK4. Furthermore, the relative contributions of site-specific phosphorylation on SPAK/OxSR1 activity in vivo remain unclear. Collectively, our in vivo data suggest that SPAK/OxSR1 serine phosphorylation plays a significant role in NCC regulation in both salt-resistant and salt-sensitive phenotypes and exhibits a sex-specific role of greater physiological importance in female versus male rats.

Further highlighting the complexity of WNK/SPAK/OxSR1 signaling, recent studies, conducted in vitro, have demonstrated that WNKs are able to form heteromultimers (19), a phenomenon that we hypothesize may occur in vivo. It has been reported that in vitro the formation of WNK heteromultimers may result in an additive, subadditive, or synergistic effect on NCC, depending on the ratio of WNK kinases versus SPAK/OxSR1 levels (19). Given the dramatically altered WNK4 expression and SPAK/OXSR1 dynamics in response to HS intake observed in SD and DSR rats versus DSS rats, and the profound sex differences observed in these parameters, we speculate that WNK heterodimers may be *1*) mediating the observed effects on NCC activity and expression observed in male SD and DSR versus male DSS rats and *2*) differentially influencing NCC activity and expression in male versus female rats.

### Study Limitations

This study’s approach to direct blood pressure measurement is a methodical limitation as animals are acutely instrumented. However, our current approach remains the only means to simultaneously assess both blood pressure and in vivo physiological activity of NCC within the same animal (35). The use of this experimental approach increases the reproducibility and rigor of our current and previously published data and supports a strong correlation between estimated in vivo NCC activity and NCC phosphorylation (17, 22). We acknowledge that our approach to estimating in vivo ENaC activity has limitations including lack of specificity for amiloride at high concentrations. Although our study demonstrates clear changes in the protein expression of kinases in the kidney by immunoblot analysis, we acknowledge that this approach may not reflect changes selective to the DCT. Furthermore, the antibody used in this study to assess WNK1 is not able to distinguish between KS-WNK1 or L-WNK1, which is a limitation of the immunoblot approach used to assess WNK1 levels. Our results demonstrate sex-specific differences in several parameters related to blood pressure and NCC regulation. Future studies, beyond the scope of the current investigations, into the mechanistic influence of male and female sex steroids on these parameters will likely reveal important insights. Furthermore, we acknowledge that the results of this study, generated in rat models, may not be directly translatable and applicable to human physiology.

### Conclusions

The present study in male and female SD, DSR, and DSS rats demonstrates sex- and strain-dependent differential NCC regulation via the canonical WNK/SPAK/OxSR1 pathway. These data provide evidence that downregulation of the NCC in response to high dietary salt intake is conserved in salt resistance irrespective of sex but that NCC activation contributes to the salt sensitivity of blood pressure in male but not in female DSS rats. The observed sex differences in the expression of WNK1 and WNK4 in male but not in female rats in salt-resistant strains highlight the complexity, and likely sex-dependent impact, of WNK signaling in NCC regulation. Furthermore, our data suggest that suppression of SPAK/OxSR1 signaling is sufficient to downregulate NCCC expression and activity in salt-resistant and salt-sensitive female rats and that this mechanism partly contributes to the lower magnitude of hypertension observed in female DSS rats. Ultimately, this work expands our understanding of sex differences in the regulatory signaling network that influences NCC-mediated Na^+^ reabsorption and the pathophysiology of the salt sensitivity of blood pressure.

## DATA AVAILABILITY

Data will be made available upon reasonable request.

## SUPPLEMENTAL DATA

10.6084/m9.figshare.23261495.v1 Supplemental Figs. S1–S3: https://doi.org/10.6084/m9.figshare.23261495.v1.

## GRANTS

This work was supported by National Institutes of Health Grants R01HL139867, R01HL141406, R01AG062515, R01AG062515-S1, and R01AG075963 and by Hevolution Foundation Grant HF-GRO-23-1199246-43 (to R.D.W.).

## DISCLOSURES

No conflicts of interest, financial or otherwise, are declared by the authors.

## AUTHOR CONTRIBUTIONS

K.K., K.M.N., F.P., J.M., and R.D.W. conceived and designed research; K.K., F.P., J.M., and R.D.W. performed experiments; K.K., K.M.N., F.P., J.M., and R.D.W. analyzed data; K.K., K.M.N., F.P., J.M., and R.D.W. interpreted results of experiments; K.K., J.M., and R.D.W. prepared figures; K.K., K.M.N., and R.D.W. drafted manuscript; K.K., K.M.N., and R.D.W. edited and revised manuscript; K.K., K.M.N., F.P., J.M., and R.D.W. approved final version of manuscript.
